# Supplementary material for: Seroconversion stages COVID19 into distinct pathophysiological states
Source: medRxiv. 2020 Dec 7:2020.12.05.20244442. Preprint. [Version 1] doi: 10.1101/2020.12.05.20244442 (PMC7743101; doi:10.1101/2020.12.05.20244442)
Supplement: 1 [file NIHPP2020.12.05.20244442-supplement-1.pdf]

medRxiv preprint doi: https://doi.org/10.1101/2020.12.05.20244442; this version posted December 7, 2020. The copyright holder for this preprint (which was not certified by peer review) is the author/funder, who has granted medRxiv a license to display the preprint in perpetuity.
It is made available under a CC-BY 4.0 International license .

SUPPLEMENTARY FIGURES

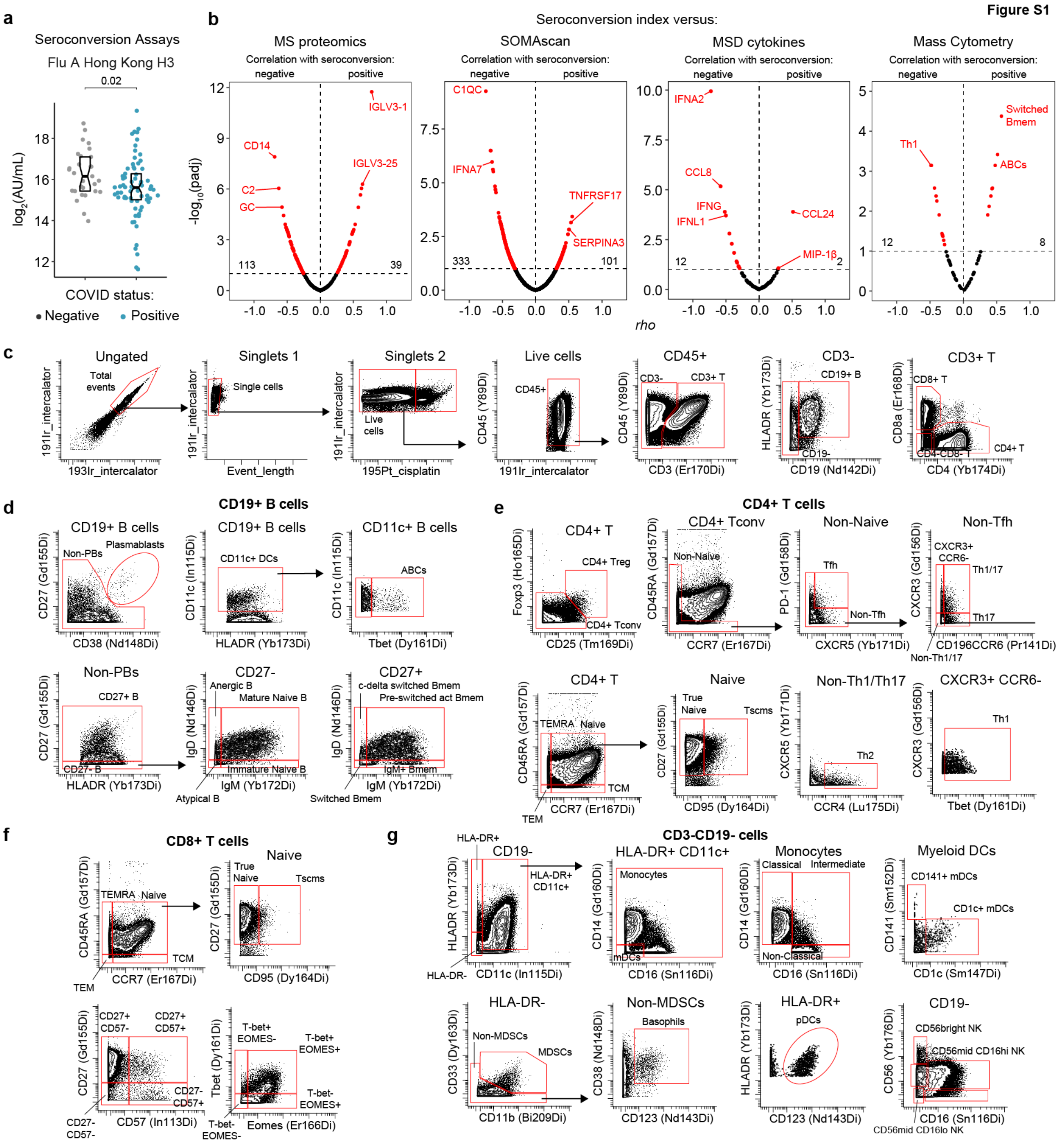

Figure S1, related to Figure 1. Biosignatures of seroconversion among hospitalized COVID19 patients. a. Meso Scale Discovery (MSD) assays show no elevation in levels of circulating antibodies against the Flu A Hong Kong H3 virus strain among COVID19 patients. Data are presented as modified Sina plots with boxes indicating median and interquartile range. Number above brackets is p-value for

medRxiv preprint doi: https://doi.org/10.1101/2020.12.05.20244442; this version posted December 7, 2020. The copyright holder for this preprint (which was not certified by peer review) is the author/funder, who has granted medRxiv a license to display the preprint in perpetuity.
It is made available under a CC-BY 4.0 International license .

Mann-Whitney test. **b.** Volcano plots for Spearman correlations between seroconversion indices and circulating plasma proteins detected by mass spectrometry (MS), SOMAscan® assays, MSD assays, as well as immune cell subsets detected by mass cytometry (MC) among all live cells. X axes show Spearman *rho* values. Y axes show $-\log_{10}$ p-values adjusted with Benjamini-Hochberg method. Dashed vertical line indicates *rho* = 0. Dashed horizontal line indicates the statistical cut off of false discovery rate (FDR)=10 (q=0.1). **c-g**. Representation of gating strategy employed during mass cytometry analysis of peripheral immune cell lineages. In (c), single live cells were gated for CD45+ staining followed by gating into T cells (CD3+), B cells (CD3- CD19+), and CD4+ and CD8+ T cells subsets. In (d), B cells were further gated into the indicated subsets. In (e) and (f), CD4+ and CD8+ T cell lineages were further characterized in the indicated subsets. Panel (g) shows gating for the indicated myeloid subsets and Natural Killer (NK) cells.

medRxiv preprint doi: https://doi.org/10.1101/2020.12.05.20244442; this version posted December 7, 2020. The copyright holder for this preprint
(which was not certified by peer review) is the author/funder, who has granted medRxiv a license to display the preprint in perpetuity.
It is made available under a CC-BY 4.0 International license .

Figure S2

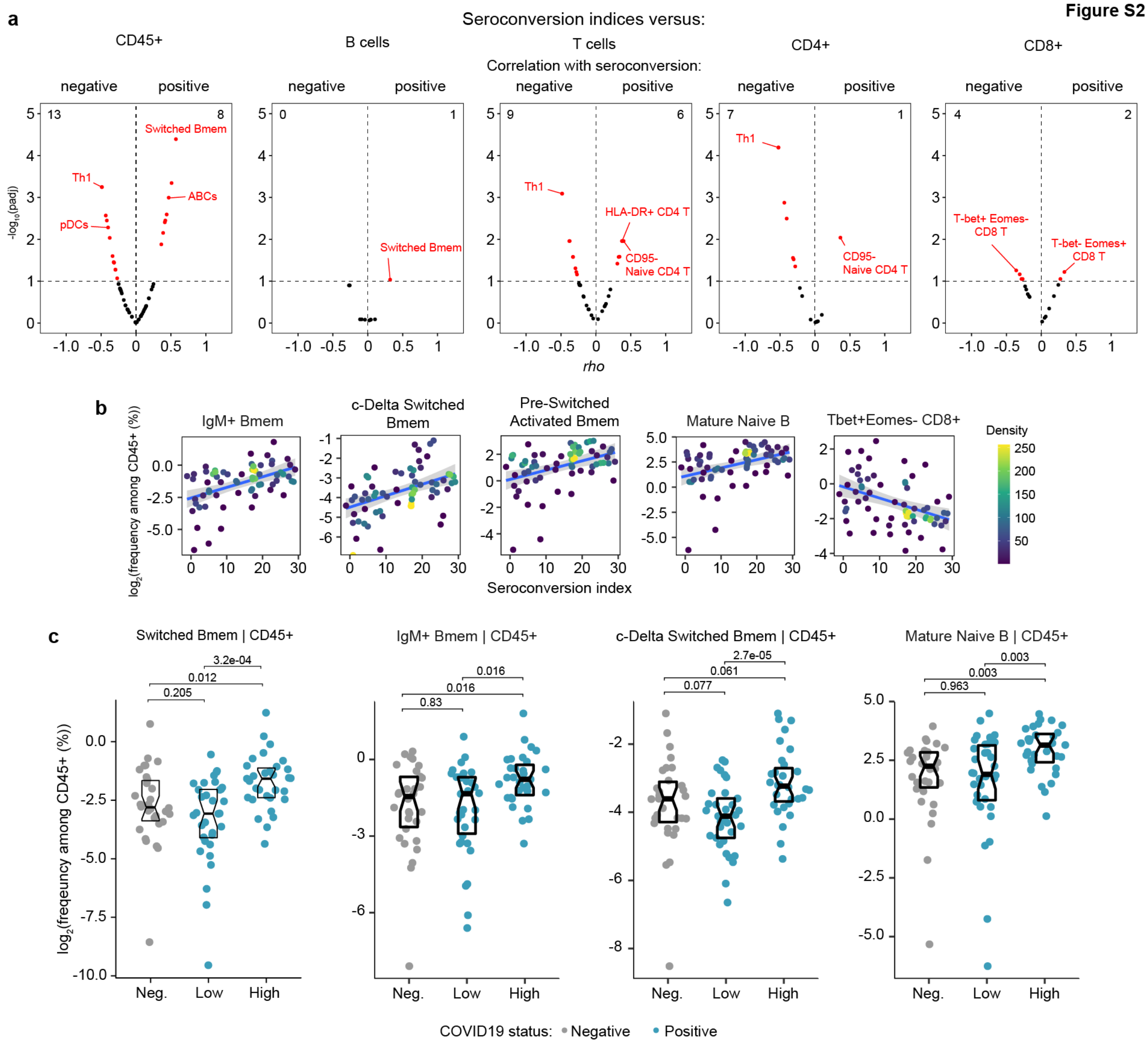

Figure S2, related to Figure 2. Immune cell signatures of seroconversion. a. Volcano plots displaying the correlations between seroconversion indices and circulating levels of immune cell subsets detected by mass cytometry (MC). X axes show Spearman *rho* values. Y axes show -$\log_{10}$ p-values adjusted with Benjamini-Hochberg method. Dashed vertical line indicates *rho* = 0. Dashed horizontal line indicates a false discovery rate (FDR) threshold of 10% (Q=0.1). Correlations were calculated for immune cell subsets measured among all live CD45+ cells (far left), all B cells, all T cells, CD4+ T cells, and CD8+ T cells (far right). **b.** Scatter plots for indicated cell types against seroconversion indices. Values shown are derived from frequency among all CD45+ cells. Points are colored by density; lines represent linear model fit with 95% confidence interval. **c.** Sina plots for

medRxiv preprint doi: https://doi.org/10.1101/2020.12.05.20244442; this version posted December 7, 2020. The copyright holder for this preprint (which was not certified by peer review) is the author/funder, who has granted medRxiv a license to display the preprint in perpetuity.
It is made available under a CC-BY 4.0 International license .

indicated immune cell types comparing controls (Negative, Neg.) to COVID19 patients divided into seroconversion low (Low) and high (High) status. Data are presented as modified Sina plots with boxes indicating median and interquartile range. Number above brackets is Q-value for Mann-Whitney tests.

medRxiv preprint doi: https://doi.org/10.1101/2020.12.05.20244442; this version posted December 7, 2020. The copyright holder for this preprint
(which was not certified by peer review) is the author/funder, who has granted medRxiv a license to display the preprint in perpetuity.
It is made available under a CC-BY 4.0 International license .

Figure S3

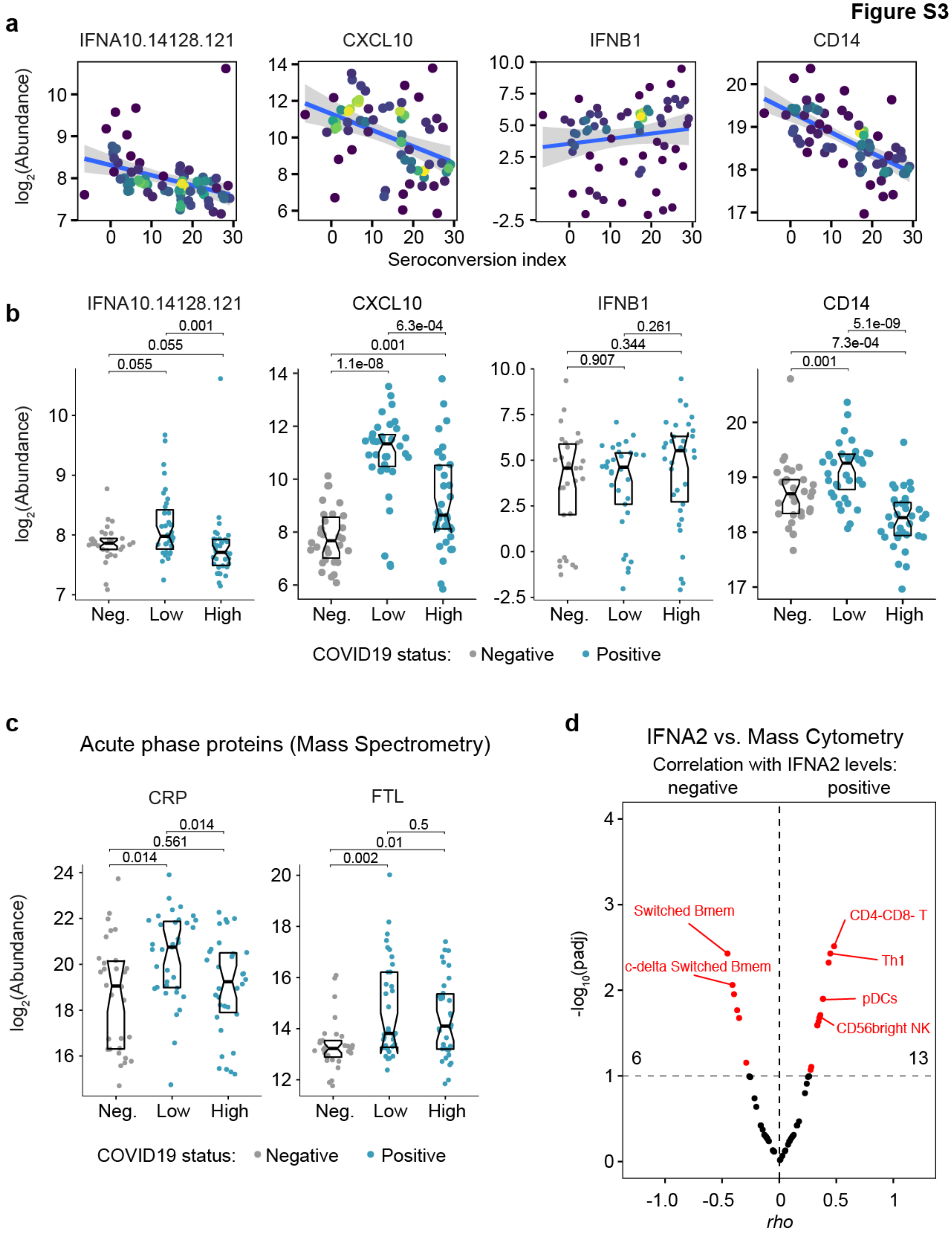

Figure S3, related to Figure 3. Seroconversion associates with differential abundance of circulating immune factors. a-b. XY scatter plots (a) and Sina plots (b) for select circulating immune factors. Points in (a) are colored by density; lines represent linear model fit with 95% confidence

medRxiv preprint doi: https://doi.org/10.1101/2020.12.05.20244442; this version posted December 7, 2020. The copyright holder for this preprint (which was not certified by peer review) is the author/funder, who has granted medRxiv a license to display the preprint in perpetuity.
It is made available under a CC-BY 4.0 International license .

interval. Data in (b) are presented as modified Sina plots with boxes indicating median and interquartile range. Number above brackets is p-value for Mann-Whitney tests. **c.** Sina plots for the acute phase proteins CRP and FTL (Ferritin Light Chain) detected by MS comparing controls (Neg., negative) to COVID19 patients divided into seroconversion low (Low) and high (High) status. Data are presented as in b. **d.** Volcano plot showing associations between circulating levels of IFNA2 measured by MSD versus immune cell subsets among all live peripheral blood mononuclear cells. X axes show Spearman *rho* values. Y axes show -$\log_{10}$ p-values adjusted with Benjamini-Hochberg method. Dashed vertical line indicates *rho* = 0. Dashed horizontal line indicates a false discovery rate (FDR) threshold of 10% (Q=0.1).

medRxiv preprint doi: https://doi.org/10.1101/2020.12.05.20244442; this version posted December 7, 2020. The copyright holder for this preprint (which was not certified by peer review) is the author/funder, who has granted medRxiv a license to display the preprint in perpetuity.
It is made available under a CC-BY 4.0 International license .

Figure S4

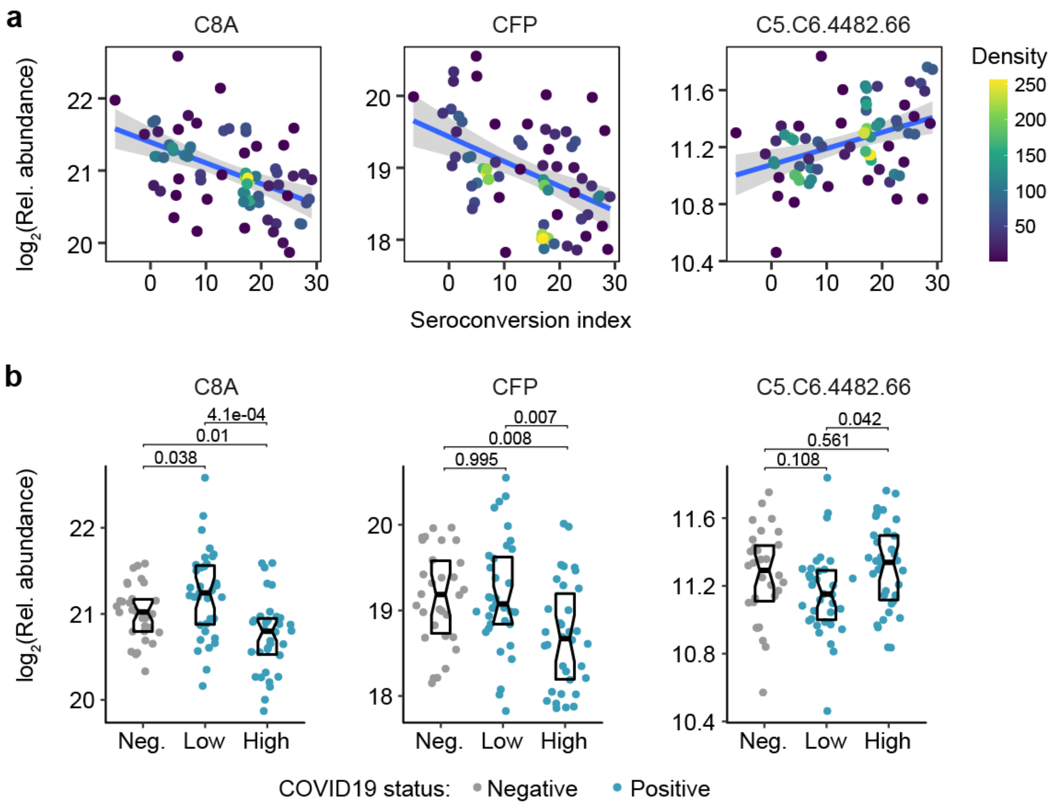

Figure S4, related to Figure 4. Seroconversion associates with decreased markers of systemic complement activation. a-b. XY scatter plots (a) and Sina plots (b) for select complement factors significantly associated, either positively or negatively, with seroconversion indices among COVID19 patients. Points in (a) are colored by density; lines represent linear model fit with 95% confidence interval. Data in (b) are presented as modified Sina plots with boxes indicating median and interquartile range comparing controls (Neg., negative) to COVID19 patients divided into seroconversion low (Low) and high (High) status. Number above brackets is p-value for Mann-Whitney tests.

medRxiv preprint doi: https://doi.org/10.1101/2020.12.05.20244442; this version posted December 7, 2020. The copyright holder for this preprint (which was not certified by peer review) is the author/funder, who has granted medRxiv a license to display the preprint in perpetuity.
It is made available under a CC-BY 4.0 International license .

Figure S5

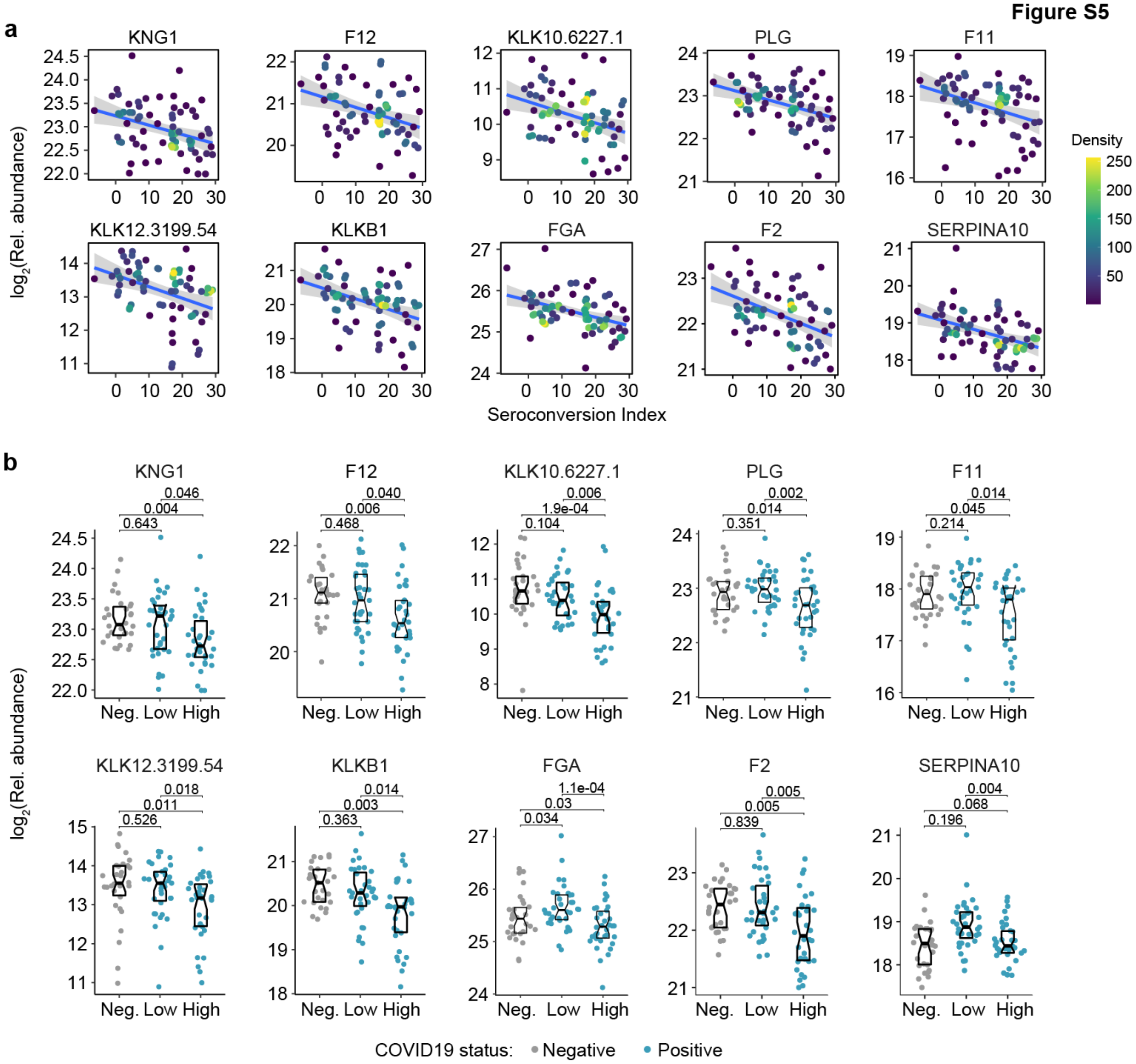

Figure S5, related to Figure 5. Seroconversion associates with remodeling of the hemostasis network. a-b. XY scatter plots (a) and Sina plots (b) for select factors involved in control of hemostasis significantly associated, either positively or negatively, with seroconversion indices among COVID19 patients. Points in (b) are colored by density; lines represent linear model fit with 95% confidence interval. Data in (b) are presented as modified Sina plots with boxes indicating median and interquartile range comparing controls (Neg., negative) to COVID19 patients divided into seroconversion low (Low) and high (High) status. Number above brackets is p-value for Mann-Whitney tests.

medRxiv preprint doi: https://doi.org/10.1101/2020.12.05.20244442; this version posted December 7, 2020. The copyright holder for this preprint
(which was not certified by peer review) is the author/funder, who has granted medRxiv a license to display the preprint in perpetuity.
It is made available under a CC-BY 4.0 International license .

Figure S6

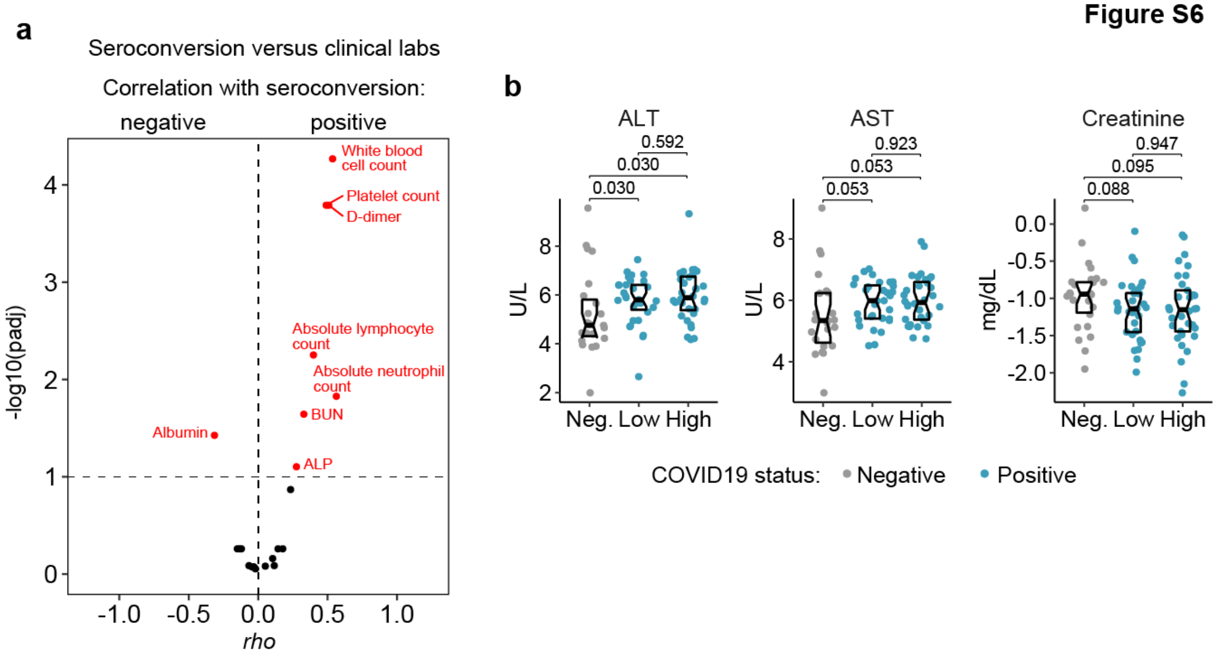

Figure S6, related to Figure 6. Seroconversion associates with recovery of blood cell counts and hypoalbuminemia. a. Volcano plot displaying the correlations between seroconversion indices and clinical laboratory values. X axes show Spearman *rho* values. Y axes show -$\log_{10}$ p-values adjusted with Benjamini-Hochberg method. Dashed vertical line indicates *rho* = 0. Dashed horizontal line indicates a false discovery rate (FDR) threshold of 10% (Q=0.1). **b.** Sina plots for select clinical laboratory values. Data in are presented as modified Sina plots with boxes indicating median and interquartile range comparing controls (Neg., negative) to COVID19 patients divided into seroconversion low (Low) and high (High) status. Number above brackets is p-value for Mann-Whitney tests.

medRxiv preprint doi: https://doi.org/10.1101/2020.12.05.20244442; this version posted December 7, 2020. The copyright holder for this preprint (which was not certified by peer review) is the author/funder, who has granted medRxiv a license to display the preprint in perpetuity.
It is made available under a CC-BY 4.0 International license .

SUPPLEMENTARY FILES LEGENDS

Supp. File S1. Cohort characteristics. Table summarizing cohort characteristics. Information pertaining less than 10% of the cohort is indicated as <10% to prevent potential reidentification.

Supp. File S2. Seroconversion versus mass spectrometry proteomics. Results of Spearman correlation analysis between seroconversion indices and proteins identified by Mass Spectrometry. Column A indicates the protein name, column B indicates the SwissProt ID, column C indicates the Spearman *rho* value, column D indicates the p value, and column E indicates the adjusted p value using the Benjamini-Hochberg method.

Supp. File S3. Seroconversion versus SOMAcan® proteomics. Results of Spearman correlation analysis between seroconversion indices and proteins identified by SOMAscan® technology. Column A indicates the SOMAmer identification number, column B indicates the target protein recognized by the SOMAmer, column C indicate the SwissProt ID, column D indicates the gene symsol, column E indicates the Spearman *rho* value, column F indicates the p value, and column G indicates the adjusted p value using the Benjamini-Hochberg method.

Supp. File S4. Seroconversion versus MSD cytokine profiling. Results of Spearman correlation analysis between seroconversion indices and cytokines, chemokines and other immune factors measured by multiplex immunoassays using Meso Scale Discovery (MSD) technology. Column A indicates the MSD analyte name, column B indicates the Spearman *rho* value, column C indicates the p value, and column D indicates the adjusted p value using the Benjamini-Hochberg method.

Supp. File S5. Seroconversion versus mass cytometry. Results of Spearman correlation analysis between seroconversion indices and immune cell subsets measured by mass cytometry (MC). Separate tabs are used for the results obtained from different parent lineages: all live cells, all CD45+ live cells, B cells, T cells, CD4+ T cells, CD8+ T cells, myeloid dendritic cells (mDCs) and monocytes. In each tab, column A indicates the population measured, column B indicates parent lineage, column C indicates the Spearman *rho* value, column D indicates the p value, and column E indicates the adjusted p value using the Benjamini-Hochberg method.

medRxiv preprint doi: https://doi.org/10.1101/2020.12.05.20244442; this version posted December 7, 2020. The copyright holder for this preprint (which was not certified by peer review) is the author/funder, who has granted medRxiv a license to display the preprint in perpetuity.
It is made available under a CC-BY 4.0 International license .

Supp. File S6. Seroconversion versus immune factors. Results of Spearman correlation analysis between seroconversion indices and circulating immune factors measured by mass spectrometry, SOMAscan® assays, or multiplex immunoassays with MSD technology. Column A indicates the analyte name in the platform, column B indicates the name used for display in the corresponding figure, column C indicates the platform used to measure the indicated analyte, column D indicates the Spearman *rho* value, column E indicates the p value, and column F indicates the adjusted p value using the Benjamini-Hochberg method.

Supp. File S7. IFNA2 versus mass cytometry. Results of Spearman correlation analysis between levels of IFNA2 measured by multiplex immunoassays using MSD technology versus immune cell subsets measured by mass cytometry. Column A indicates the immune cell population among all live cells, column B indicates the lineage used to calculate cell frequencies, column C indicates the Spearman *rho* value, column D indicates the p value, and column E indicates the adjusted p value using the Benjamini-Hochberg method.

Supp. File S8. Seroconversion versus complement. Results of Spearman correlation analysis between seroconversion indices and components of the complement pathways measured by mass spectrometry or SOMAscan® assays. Column A indicates the unique identifier within the platform, column B indicates the name use for display in the corresponding figure, column C indicates the platform used to measure the indicated analyte, column D indicates the Spearman *rho* value, column E indicates the p value, and column E indicates the adjusted p value using the Benjamini-Hochberg method.

Supp. File S9. Seroconversion versus hemostasis network. Results of Spearman correlation analysis between seroconversion indices and factors involved in control of hemostasis measured by mass spectrometry or SOMAscan® assays. Column A indicates the unique identifier within the platform, column B indicates the name use for display in the corresponding figure, column C indicates the platform used to measure the indicated analyte, column D indicates the Spearman *rho* value, column E indicates the p value, and column E indicates the adjusted p value using the Benjamini-Hochberg method.

medRxiv preprint doi: https://doi.org/10.1101/2020.12.05.20244442; this version posted December 7, 2020. The copyright holder for this preprint (which was not certified by peer review) is the author/funder, who has granted medRxiv a license to display the preprint in perpetuity.
It is made available under a CC-BY 4.0 International license .

Supp. File S10. Seroconversion versus clinical laboratory values. Results of Spearman correlation analysis between seroconversion indices and clinical laboratory values closest to the time of the research blood draw. Column A indicates the clinical laboratory parameter, column B indicates the Spearman *rho* value, column C indicates the p value, and column D indicates the adjusted p value using the Benjamini-Hochberg method.

Supp. File S11. Mass cytometry antibody table. List of antibodies used in mass cytometry. Column A indicates the antibody target, column B indicates the element conjugated to the antibody, column C indicates the mass of the element, column D indicates the manufacturer, column E indicates the catalog number, column F indicates the clone number, and column G indicates the type of stain protocol used (fixed, live or fixed with permeabilization).

Supp. File S12. Immune cell type definition. List of immune cell subsets defined by mass cytometry. Column A indicates the population identified, column B indicates definition based on gating strategy employed, and column C indicates the parent lineage.
